# Transfer and generalization of learned manipulation between unimanual and bimanual tasks

**DOI:** 10.1038/s41598-021-87988-0

**Published:** 2021-04-22

**Authors:** Trevor Lee-Miller, Marco Santello, Andrew M. Gordon

**Affiliations:** 1 Department of Biobehavioral Sciences, Teachers College, Columbia University, Box 93, New York, NY 10027 USA; 2 School of Biological and Health Systems Engineering, Arizona State University, Tempe, AZ 85287-9709 USA

**Keywords:** Motor control, Sensorimotor processing

## Abstract

Successful object manipulation, such as preventing object roll, relies on the modulation of forces and centers of pressure (point of application of digits on each grasp surface) prior to lift onset to generate a compensatory torque. Whether or not generalization of learned manipulation can occur after adding or removing effectors is not known. We examined this by recruiting participants to perform lifts in unimanual and bimanual grasps and analyzed results before and after transfer. Our results show partial generalization of learned manipulation occurred when switching from a (1) unimanual to bimanual grasp regardless of object center of mass, and (2) bimanual to unimanual grasp when the center of mass was on the thumb side. Partial generalization was driven by the modulation of effectors’ center of pressure, in the appropriate direction but of insufficient magnitude, while load forces did not contribute to torque generation after transfer. In addition, we show that the combination of effector forces and centers of pressure in the generation of compensatory torque differ between unimanual and bimanual grasping. These findings highlight that (1) high-level representations of learned manipulation enable only partial learning transfer when adding or removing effectors, and (2) such partial generalization is mainly driven by modulation of effectors’ center of pressure.

## Introduction

Skilled object manipulation is accomplished through the sensorimotor coordination of effector kinetics and kinematics to object properties and task goal^1–8^. Effector kinetics (digit forces) and kinematics (digit placement) have been shown to be controlled in anticipation of an external object torque through a continuum of control between grasp kinetics and kinematics (for review^5^). Specifically, when digit placement is unconstrained, learned manipulation relies on a digit force-to-placement coordination to successfully perform the task^2,9^. When lifting objects with different centers of mass^10–12^, learned manipulation involves the modulation of digit forces and placement to generate a compensatory torque to prevent object roll^2,13^. Digit centers of pressure and load forces are modulated by placing the digit on the side of the center of mass higher and applying more load force at that digit. Covariation of digit forces-to-placement provides evidence of a high-level representation of learned compensatory torque that drives the flexible coordination where digit forces are selected based on the feedback of digit placement^2,14^. Aside from two-digit precision grasping, we have recently shown that digit force-to-placement modulation is a general feature of dexterous manipulation that is also found in whole-hand^15^ and bimanual^16^ manipulation.

A high-level representation was shown to generalize when adding or removing degrees of freedom, between two- to three-digit grasps, where learned manipulation resulted in similar torques before and after transfer^17^. In contrast, learned compensatory torque does not transfer across hands^18–20^, or after switching hands with object rotation, in an attempt to maintain the mirrored dynamics between the hands^18,19,21^. Thus, learned manipulation of object torque seems to be specific to the frame of reference between the object and body^22^. The successful transfer between changing degrees of freedom of the same effector (two- to three-digits) and the finding of high-level representation enabling digit force-to-position coordination in both unimanual and bimanual grasps raises the following question: Can high-level representations of dexterous manipulation generalize after adding or removing effectors while maintaining the same frame of reference?

To address this question, we examined the extent to which the ability to generate a compensatory torque could be transferred in response to changing the grasp context, i.e., the effector(s) previously used to learn the manipulation, and whether learning transfer would be sensitive to whether effectors are added or removed relative to the previous context. We used two transfer conditions: (1) unimanual to bimanual manipulation, and (2) bimanual to unimanual manipulation and quantified learning transfer by measuring the direction and magnitude of compensatory torque before and after changing grasp context. Similar to a previous study, generalization has been shown when adding or removing digits to the grasp^17^. Additionally, in our two transfer conditions (unimanual to bimanual grasp, and vice-versa), the frame of reference relative to the object, i.e., the direction of the external torque in an object frame of reference, in which compensatory torque was learned in the pre-transfer grasp is the same as the one in the post-transfer grasp. As noted above, congruency in the frame of reference has been identified as an important determinant for transferring learned manipulation. Therefore, we hypothesized successful generalization when adding or removing effectors.

## Results

**Figure 1:**
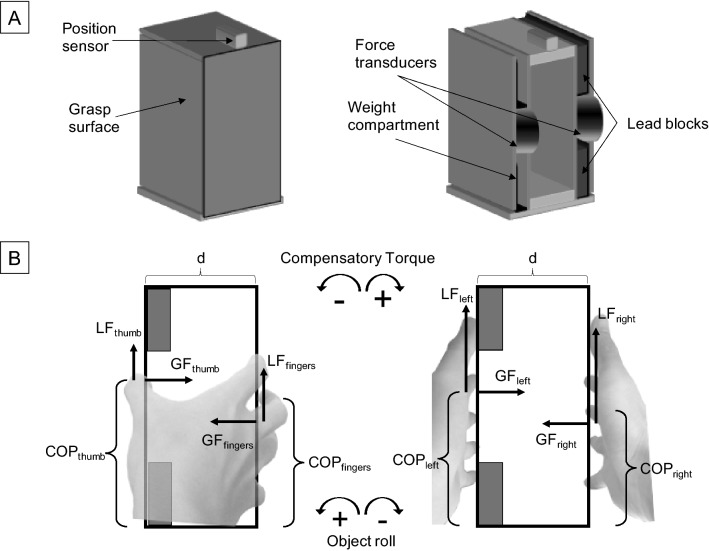
Experimental Apparatus. (**A**) Physical appearance of the object as presented to the participants and the inner components. (**B**) Schematic layout of the grasped object showing the total forces produced by the digits on each side and the direction of resultant compensatory torque and object roll.

Participants were assigned into two groups of 10 in a pseudo-random order, with each group performing lifts in one of the transfer conditions as described in Table 1. Lifts were performed on a visually symmetrical box (height, width, depth = 16.5, 8.1, 8.5 cm) while the obscured center of mass was either on the left or right (Fig. 1). We recorded the forces and torques on each side of the grasp surface, which allowed us to measure the effector grip (GF) and load force (LF), and the effector center of pressure (COP). A measure of the difference between the left and right grasp surface was used for LF and COP. Compensatory torque (Tcom) was calculated from the combined torques generated from COP_diff_ and GF, and LF_diff_. Specifically, the GF of the digits act on the COPs to generate the normal component of the total torque (GF × COP_diff_). Additionally, LF generate the tangential component of the total torque by acting on the width between the grasp surfaces (8.1 cm) as the moment arm. Thus, multiplying LF_diff_ by half object width (4.05 cm) gives the tangential torque component.

**Table 1:**
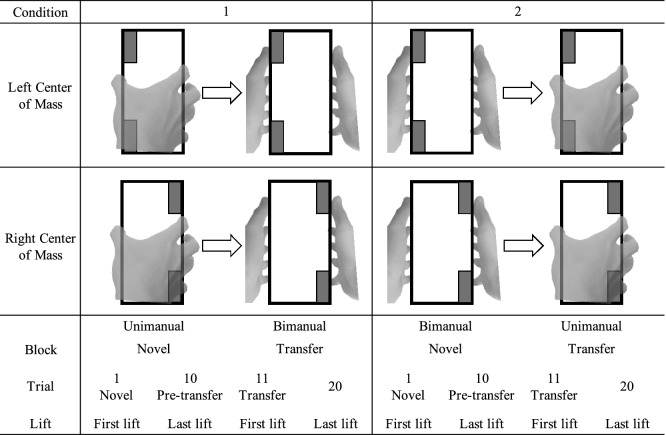
Grasp order of each condition. Breakdown of the direction of transfer and direction of torque used for each condition. 10 participants performed lifts in condition 1 and another 10 participants performed lifts in condition 2. Center of mass order was counterbalanced. Trial 1 for both conditions represents participants’ first lift with the object. Trial 11 represents the first post-transfer lift. Trials 10, and 20 represent the last lift in either grasp type.

### Learned generation of Tcom differs between unimanual and bimanual grasps

**Figure 2:**
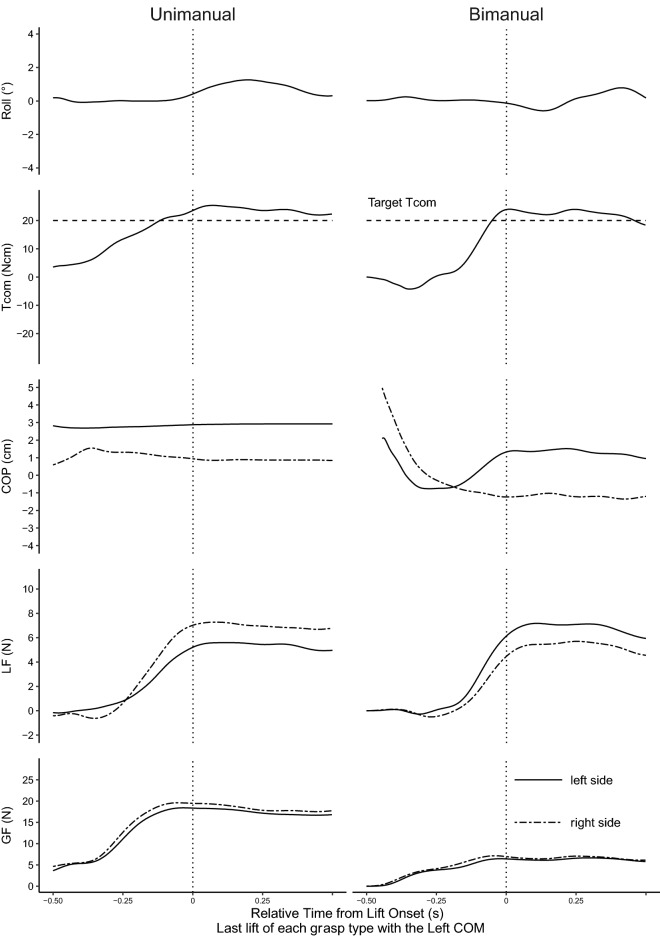
Representative plots for the left center of mass (COM). Traces for the learned trials of all measures for the unimanual and bimanual grasps. Tcom = compensatory torque, COP = center of pressure, LF = load force, GF = grip force. Vertical dotted lines represent time at lift onset.

**Figure 3:**
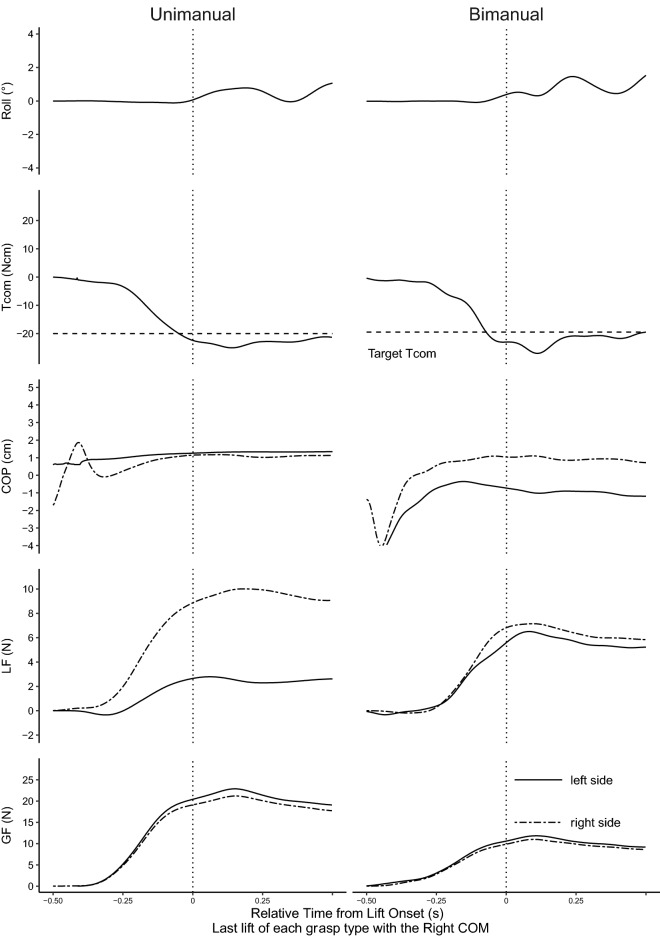
Representative plots for the right center of mass (COM). Traces for the learned trials of all measures for the unimanual and bimanual grasps. Tcom = compensatory torque, COP = center of pressure, LF = load force, GF = grip force. Vertical dotted lines represent time at lift onset.

On the last lift in all grasp types, all participants performed the task successfully through the minimization of peak roll and generation of the appropriate Tcom. However, even though Tcom was similar across the different grasps, the way in which Tcom was generated differed between the grasps. Figures 2 and 3 show the representative traces of object roll, Tcom, COP, LF, and GF on the last lift. Minimization of peak roll (> − 2°, or < 2°) and generation of the appropriate Tcom (~ ± 20 Ncm) was seen for all grasps, with both being correlated measures of task performance. However, the grasp type affected the way Tcom was generated. For unimanual COP, learned manipulation of the left COM was characterized by generally higher thumb (left side) COP than that of the fingers (right side). For the right COM, COPs were mainly collinear. In bimanual grasps, left hand COP was higher than the right hand for left COM, whereas right hand COP was higher for right COMs. Thus, on the last lifts, COP_diff_ between unimanual and bimanual grasps did not differ for the left COM (condition 1, *p* = 0.60; condition 2, *p* = 1.00) but differed for the right COM (condition 1, *p* < 0.001; condition 2, *p* < 0.001). Unimanual LFs showed larger LFs of the fingers (right side) with larger differences when COM was on the right (Fig. 3). Bimanual grasps showed larger LFs of the left compared to the right hand for left COMs and larger right hand LFs for right COMs. Unimanual and bimanual LFs on the last lift differed for both COMs (left COM, *p* < 0.001; right COM, *p* = 0.001) It should be noted that the COP and LF measured in this study is the equivalent of all the digits on the corresponding side. Unimanual GFs were larger than bimanual GFs (condition 1, *p* < 0.001; condition 2, *p* < 0.001).

Overall, in learned unimanual grasping, the COP of the thumb was either collinear or higher than the equivalent COP of the fingers while the combined LF of the four fingers was always larger than the LF of the thumb. The magnitude of these differences was determined by the COM of the object. For learned bimanual grasps, COP and LF of the hand were higher and larger on the side of the COM.

### Bimanual performance after unimanual to bimanual transfer half that of pre-transfer lifts for both left and right COM

To determine the outcome of transfer, the results of the first transfer lifts were compared to pre-transfer lifts and novel lifts in the respective grasp types. In this section, we highlight the results of the unimanual to bimanual transfer trials and compare these accordingly. Results of the bimanual to unimanual transfer will be discussed in the next section.

**Figure 4:**
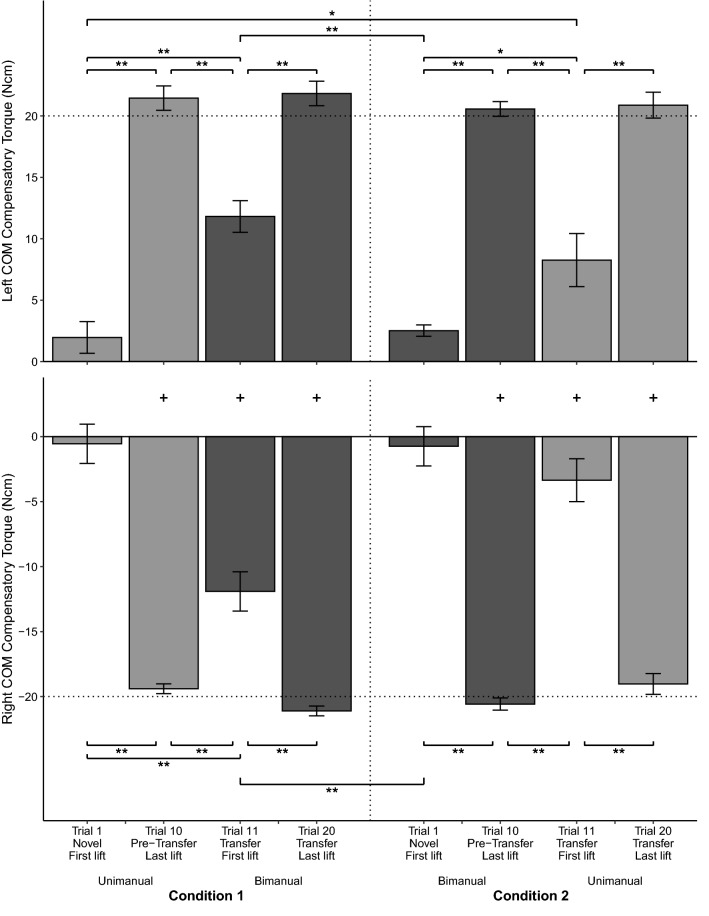
Results for Compensatory Torque (Tcom) across all conditions. Average Tcom for the left COM (top panel) and right COM (bottom panel) for the novel first trial, pre-transfer trial 10, transfer trial 11, and trial 20 (± s.e.m). Light grey bar indicates Tcom for the right-hand unimanual trials. Dark grey bar indicates Tcom for the bimanual trials. Error bars indicate the standard error of the mean. Horizontal lines indicate the target Tcom while the vertical line separates condition 1 (left panel) from condition 2 (right panel). *p < .05, **p < .001, + *p* < .05 between the left and right COM.

**Figure 5:**
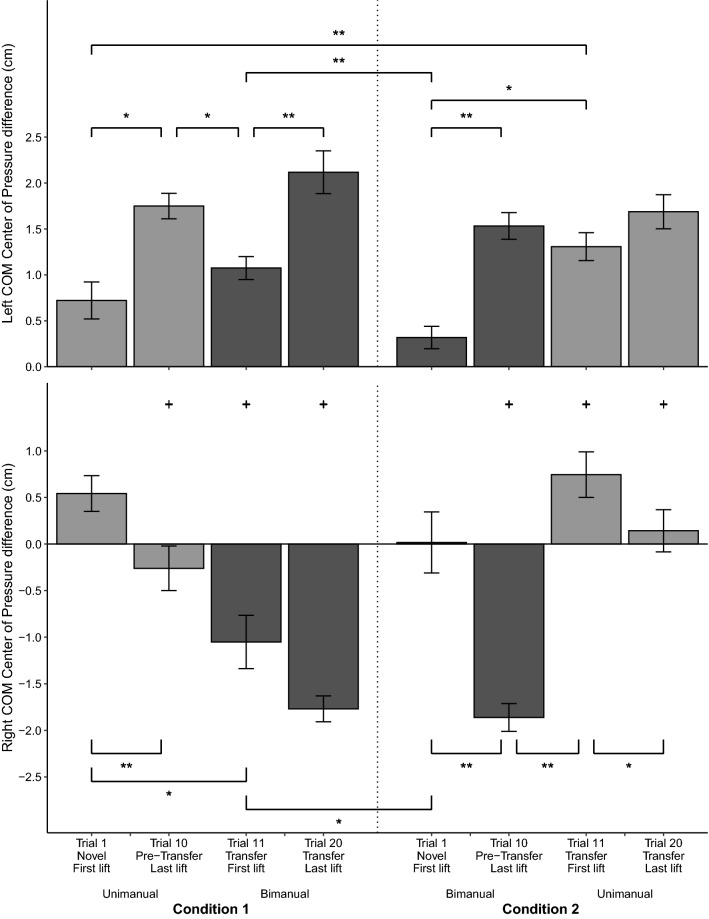
Results for Center of Pressure difference (COP_diff_) across all conditions. Average COP_diff_ for the left COM (top panel) and right COM (bottom panel) for the novel first trial, pre-transfer trial 10, transfer trial 11, and trial 20 (± s.e.m). Light grey bar indicates COP_diff_ for the right-hand unimanual trials. Dark grey bar indicates COP_diff_ for the bimanual trials. Error bars indicate the standard error of the mean. The vertical line separates condition 1 (left panel) from condition 2 (right panel). *p < .05, **p < .001, + *p* < .05 between the left and right COM.

**Figure 6:**
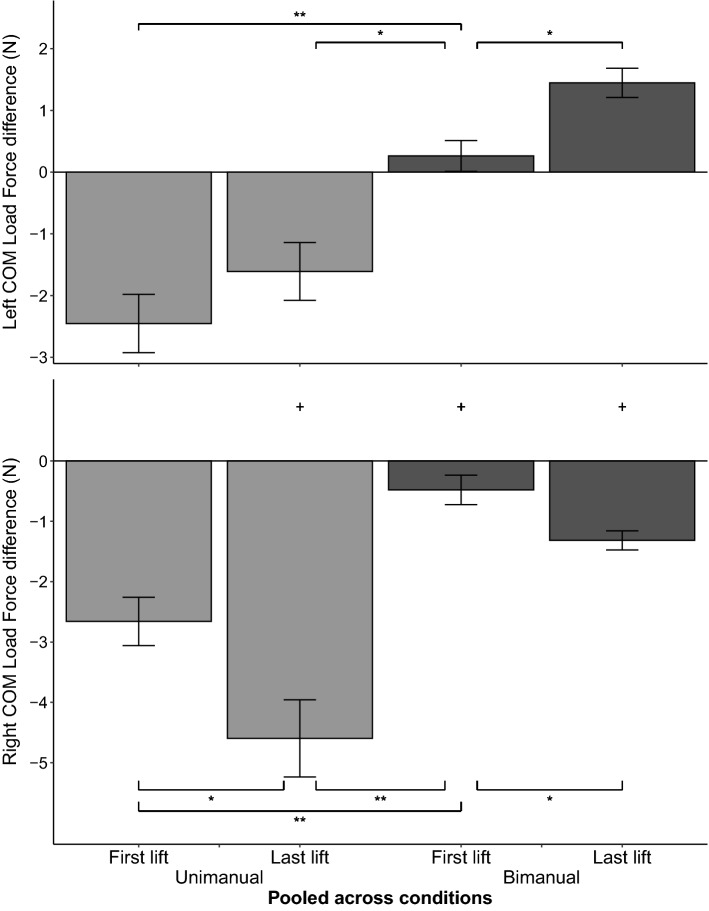
Results for Load Force difference (LF_diff_). Average LF_diff_ for the left COM (top panel) and right COM (bottom panel) for the first and last lifts in each grasp type (± s.e.m). Results were pooled across conditions after no significant effect was found between conditions. Light grey bar indicates LF_diff_ for the right-hand unimanual trials. Dark grey bar indicates LF_diff_ for the bimanual trials. Error bars indicate the standard error of the mean. *p < .05, **p < .001, + *p* < .05 between the left and right COM.

**Figure 7:**
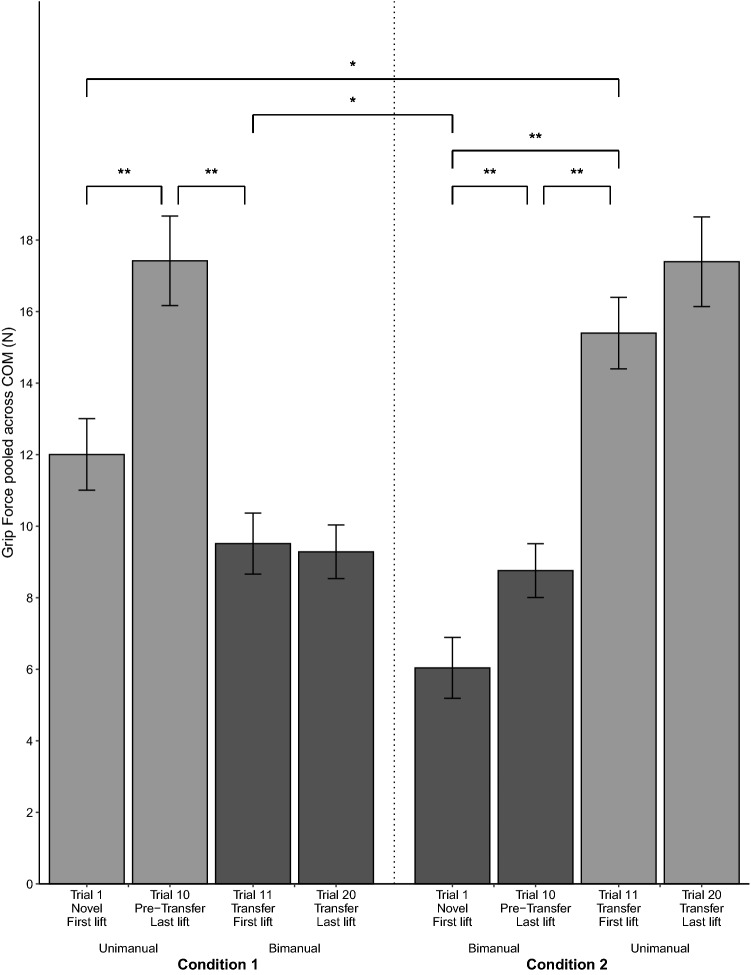
Results for Grip Force (GF) across the conditions. Average GF for condition 1(left panel) and condition 2 (right panel) for the novel first trial, pre-transfer trial 10, transfer trial 11, and trial 20 (± s.e.m). Results were pooled across COM after no significant effect was found between the left and right COM. Light grey bar indicates GF for the right-hand unimanual trials. Dark grey bar indicates GF for the bimanual trials. Error bars indicate the standard error of the mean. *p < .05, **p < .001.

After transfer, bimanual Tcom was applied in the appropriate direction to prevent object roll (condition 1). However, the generated Tcom on the transfer lift was smaller than the Tcom of the unimanual pre-transfer lift. Results show that for the unimanual to bimanual condition 1, Tcom differed between (1) pre-transfer and transfer trials, (2) left and right COM, and (3) the first and last lifts (significant interaction between trial, COM, and condition, *F*(3,54) = 36.18, *p* < 0.001, η_p_^2^ = 0.67). These results also indicated that across conditions, bimanual grasps differed between first lifts of the transfer trials (condition 1) and the first novel lifts (condition 2). Figure 4 shows the Tcom for the novel trial 1, pre-transfer trial 10, transfer trial 11, and trial 20 for the left and right COM across both grasps in conditions 1 and 2. Post hoc tests showed that for the Tcom in condition 1, the first bimanual transfer lift was smaller than the unimanual pre-transfer lift for both COMs (left COM, *p* < 0.001; right COM, *p* = 0.001). However, across conditions, bimanual Tcom after transfer was larger than Tcom for the first novel bimanual lift (condition 2) for both COMs (left COM, *p* < 0.001; right COM, *p* < 0.001). Comparing between COMs revealed that bimanual Tcom differed between the left and right COM for all except the first novel lifts (Fig. 4 ‘+’ sign) further showing that transfer results in Tcom generation in the appropriate direction (condition 1, transfer trial 11, *p* < 0.001; condition 1, transfer trial 20, *p* < 0.001; condition 2, novel trial 1, *p* = 0.054; condition 2, pre-transfer trial 10, *p* < 0.001). However, regardless of whether the lifts were novel or transfer, the first bimanual lifts had a smaller Tcom than the last bimanual lifts (condition 1, left COM, *p* < 0.001; condition 1, right COM, *p* < 0.001; condition 2, left COM, *p* < 0.001; condition 2, right COM, *p* < 0.001). Figure 5 shows the results for COP_diff_. COP_diff_ differed between (1) unimanual pre-transfer and bimanual transfer trials in condition 1, (2) left and right COM, (3) the first and last lifts, and (4) the bimanual transfer lift in condition 1 and novel bimanual first lift in condition 2 (significant interaction between trial, COM, and condition, *F*(3,54) = 7.33, *p* < 0.001, η_p_^2^ = 0.29). Post hoc tests revealed similar trends as Tcom. Specifically, bimanual transfer (condition 1) resulted in COP of the hand on the side of the COM being modulated higher than COP of the other hand compared to the first bimanual novel lift that had near collinear COPs (condition 2) for both COMs (left COM, *p* < 0.001; right COM, *p* = 0.025). Contrary to COP_diff_, LF_diff_ did not differ across conditions (only significant interaction between COM and trial, *F*(3,54) = 9.81, *p* < 0.001, η_p_^2^ = 0.35). Figure 6 thus shows the results of LF_diff_ averaged across both conditions. Results show that for bimanual LF_diff_, first lifts were near zero and that LF modulation was significant by the last lift (left COM, *p* = 0.039; right COM, *p* = 0.035). Results for bimanual GF showed that bimanual GF did not differ by COM but that GF was lower during the first novel lift (condition 2) compared to the first transfer lift (condition 1) and the last lifts (significant interaction between trial and condition *F*(3,54) = 5.94, *p* < 0.001, η_p_^2^ = 0.25). Figure 7 shows the results for GF pooled across COM. Additionally, bimanual GFs were lower than unimanual GFs (condition 1, *p* < 0.001; condition 2, *p* < 0.001). Results of bimanual digit placement (DP) showed that DP did not change across the lifts and only differed by digit (main effect of digit, *F*(1.74, 20.83) = 393.4, *p* < 0.001, η_p_^2^ = 0.97).

Taken together, our results show that after unimanual to bimanual transfer (adding effectors), participants attempted to minimize object roll by applying Tcom in the appropriate direction to counter the external torque on the object. Tcom was generated through COP modulation and larger GF for both centers of mass. However, although Tcom was generated in the correct direction, its magnitude was smaller than required to counter the external torque.

### Unimanual performance after bimanual to unimanual transfer half that of pre-transfer lifts for only the left COM

Results also showed that performance after transfer to a unimanual grasp was similar to that after bimanual transfer: Tcom was applied in the appropriate direction to counter object roll on the first transfer lift but was smaller than Tcom on the pre-transfer lift. However, this was only seen for the left COM, i.e., COM on the thumb’s side. Post hoc tests showed that for unimanual grasps, Tcom differed between (1) the pre-transfer bimanual and unimanual transfer lift (condition 2) for both COMs (left COM, *p* < 0.001; Right COM, *p* < 0.001), (2) novel unimanual lifts (condition 1) and unimanual transfer lifts (condition 2) for the left COM (*p* = 0.011), (3) left and right COM for all except the novel first lift (condition 1, novel trial 1, *p* = 0.14; condition 1, pre-transfer trial 10, *p* < 0.001; condition 2, transfer trial 11, *p* < 0.001; condition 2, transfer trial 20, *p* < 0.001), and (4) the first and last lifts in both conditions (condition 1, left COM, *p* < 0.001; condition 1, right COM, *p* < 0.001; condition 2, left COM, *p* < 0.001; condition 2, right COM, *p* < 0.001) (Fig. 4). Results for COP_diff_ showed similar unimanual transfer to bimanual pre-transfer COP_diff_ (Fig. 5 condition 2), but only for the left COM (left COM, *p* = 1.00, right COM, *p* < 0.001). For both novel and transfer trials, regardless of the COM, unimanual first lifts resulted in higher thumb COPs than finger COPs. However, only after transfer in the left COM was unimanual COP_diff_ similar between the first and last lifts (*p* = 0.71). Additionally, COP_diff_ showed higher thumb COPs after transfer in the left COM compared to novel unimanual lifts (*p* = 0.03) and compared to transfer in the right COM (*p* = 0.003). As mentioned, LF_diff_ did not differ between the novel and transfer conditions but differed between the left and right COM for last lifts (*p* = 0.001) and the first and last lift of the right COM (*p* = 0.006). Thus, LF_diff_ did not contribute to the difference of Tcom between the novel and transfer lifts. As mentioned earlier, for all unimanual lifts, LFs of the fingers were larger than LFs of the thumb (Fig. 6). Unimanual GF did not differ across COM with GF of the novel first lift being smaller than GF of the transfer lifts (*p* = 0.03). Analysis of DP showed that DP did not differ by condition but differed across COM and lifts (significant interaction between COM, lift, and digit, *F*(1.92,30.66) = 3.23, *p* = 0.018, η_p_^2^ = 0.17). Post hoc tests showed that on the last lift, thumb placement was 1 cm higher for the left than the right COM (*p* = 0.004).

Taken together, our results show that after bimanual to unimanual transfer (removing effectors), participants attempted to minimize object roll by applying Tcom in the appropriate direction to counter the object’s external torque, but only when object COM was on the side of the thumb. Tcom was generated by COP modulation and larger GFs. However, although Tcom was generated in the correct direction, the magnitude was smaller than the required Tcom.

### Positive transfer due to modulation of COP difference and GF

**Figure 8:**
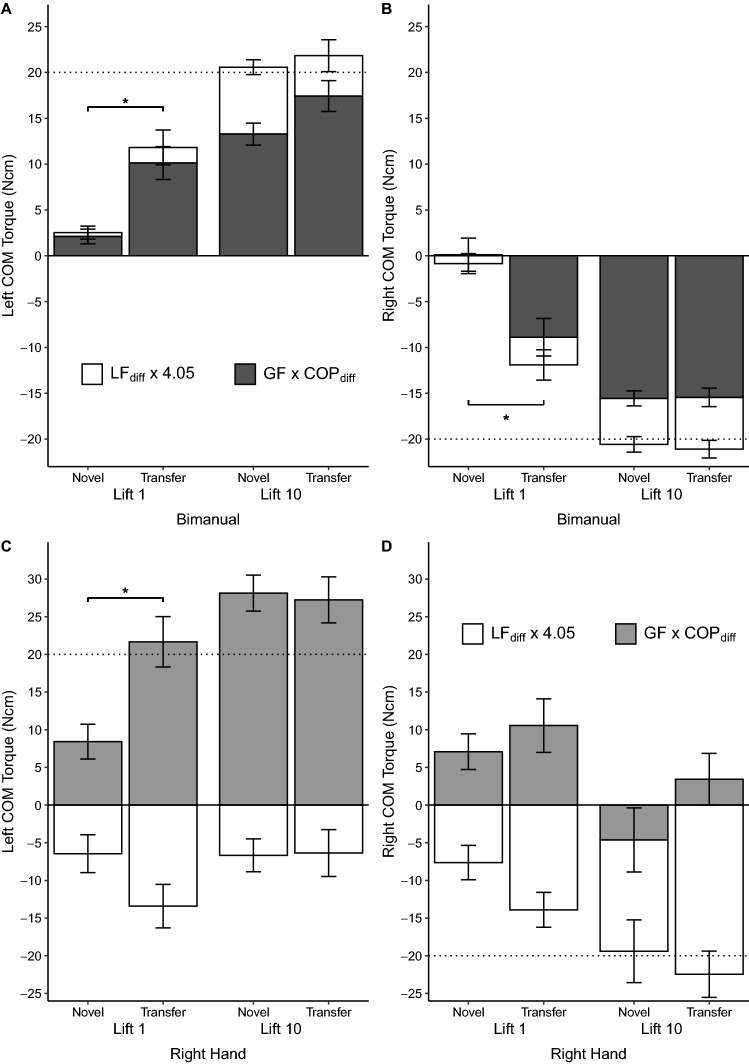
Grasp contribution of torque components to Tcom. (**A**, **B**) Stacked bar graph showing how LF_diff_ and GF with COP_diff_ affected Tcom for lift 1 and 10 of the novel and transfer conditions for the left and right COM during bimanual grasps. (**C, D**) Stacked bar graph showing how LF_diff_ and GF with COP_diff_ affected Tcom for lift 1 and 10 of the novel and transfer conditions for the left and right COM during right hand grasps. Error bars indicate the standard error of the mean. Horizontal dotted line indicates target Tcom. LF_diff_ did not differ across conditions. *p < .05 for GF × COP_diff_.

Figure 8 shows the relative contribution of both torque components to the resultant Tcom in each of the grasp types across lifts 1 and 10 for all conditions. As mentioned above, LF_diff_ did not show any significant differences between conditions. However, the normal torque component, GF × COP_diff_, showed a difference between conditions (trial, COM, condition interaction, *F*(3,54) = 11.10, *p* < 0.001, η_p_^2^ = 0.38). For bimanual grasps, transfer resulted in a larger normal torque component (GF × COP_diff_) compared to the novel first lift (left COM, *p* = 0.001; right COM, *p* = 0.004). The normal torque component at lift 1 was thus closer to the target Tcom after unimanual to bimanual transfer compared to novel lifts for both left and right COM. For unimanual grasps, transfer resulted in a higher normal torque component compared to novel lifts only for the left COM (left COM, *p* = 0.004; right COM, *p* = 0.43). Thus, bimanual to unimanual transfer of the right hand was a result of GF × COP_diff_ modulation but only when the COM was on the side of the thumb.

## Discussion

We found post-transfer performance improvement of learned manipulations compared to novel lifts of the same grasp, but poorer performance relative to the pre-transfer lift. Specifically, compensatory torque in the post-transfer lift was exerted in the appropriate direction, but of insufficient magnitude to fully counter the external torque, hence minimizing object roll to a lesser degree. Such partial generalization of learned dexterous manipulation occurred when adding effectors, i.e., when switching from unimanual to bimanual grasp, regardless of the object center of mass. Partial generalization was also revealed when removing effectors from a bimanual to unimanual grasp but only when the center of mass was on the thumb side for the unimanual grasp (left center of mass). The partial generalization of compensatory torque was driven mainly by the post-transfer modulation of effector center of pressure, leading to non-collinear centers of pressure on the left versus right grasp surface, and larger grip forces. In contrast, when effectors were removed, i.e., when switching from bimanual to unimanual grasp and the object’s center of mass was on the finger side, no learning transfer occurred. Adding an effector (unimanual to bimanual grasp) would require translating the representation learned using one hand into motor commands that now have to be shared between two hands. In contrast, removing an effector (bimanual to unimanual grasp) would require the opposite process, i.e., using the representation learned by coordinating the action of two hands to generate motor commands directed to only one hand. Despite the fact that the object frame of reference was invariant in both transfer directions, the finding of partial generalization underscores the complexity and limitations of the central nervous system in flexibly adapting high-level representations to drastic changes in low-level control elements, i.e., muscles, joints, digits and hands.

Our findings raise two questions: (1) why was generalization partial? and (2) why was partial generalization when removing effectors only seen when the object’s center of mass was on the thumb side? We suggest that a possible answer to both of these questions lies in transfer driving effector center of pressure modulation. We discuss the implications of generalization of high-level internal representations and their limitations on enabling learning transfer.

### Characteristics of torque generation in the partial generalization of learned manipulation

The partial generalization revealed by the present study is influenced by the idiosyncratic characteristics of unimanual and bimanual grasps. As such, in order to understand generalization further, we must first examine the characteristics of both grasp types as they pertain to learned manipulation. The modulation and covariation of effector center of pressure and load forces, for compensatory torque generation, indicates a high-level representation of learned manipulation and this is a general feature for both unimanual and bimanual grasps^2,15,16^. However, within each grasp type, we have shown certain idiosyncratic characteristics. For two-digit, three-digit, and bimanual grasping, center of pressure and load forces are modulated according to the direction of the external torque. Successful torque generation is thus achieved through modulation of effector’s center of pressure and load forces: both of these variables are higher and larger, respectively, on the side of the object’s center of mass. However, for five-digit unimanual grasping, the center of pressure of the thumb is either higher or collinear to the equivalent center of pressure of the fingers while finger load forces are always larger regardless of object center of mass^15^. Learned manipulation thus involves the modulation of effector center of pressure and load forces that follow these different characteristics for unimanual versus bimanual grasps.

Based on the above considerations, partial generalization could be due to either (1) the partial generation of pre-transfer torque characteristics, or (2) the partial generation of torque characteristics of the post-transfer grasp type. In the former scenario, the center of pressure and load force characteristics of a post-transfer bimanual grasp would be similar to the pre-transfer unimanual grasp characteristics and vice versa for a post-transfer unimanual grasp. In the latter scenario, the characteristics of a post-transfer bimanual grasp would be similar to learned manipulation in a bimanual grasp while post-transfer unimanual characteristics follow learned unimanual characteristics. By examining the results of transfer, we can determine which of these scenarios would best explain the partial generalization. Table 2 shows the characteristics of center of pressure and load force across the four context switch conditions (adding or removing effectors with left or right center of mass). Two conclusions can be drawn from these results: (1) partial transfer was a result of center of pressure characteristics that were similar to learned manipulation in the post-transfer grasp type (scenario 2), and (2) load force difference was unaffected by transfer. For transfers of an object with a left center of mass, both pre- and post-transfer centers of pressure involved higher left side centers of pressure, which could indicate scenario 1. However, for unimanual to bimanual transfer with a right center of mass, the pre-transfer unimanual centers of pressure were collinear while post-transfer bimanual showed higher right side center of pressure. Similarly, bimanual to unimanual transfer with a right center of mass did not lead to similar pre-transfer bimanual and unimanual transfer centers of pressure. Thus, generalization did not involve an attempt to mimic the pre-transfer grasp center of pressure (scenario 1), but rather an attempt to generate torque through center of pressure difference modulation similar to the characteristics of the post-transfer grasp (scenario 2). The partial generation of torque after transfer could consequently be explained by (1) the lack of load force difference in the direction of torque generation, or (2) the lack of load force modulation and insufficient center of pressure modulation in the direction of torque generation. Our results showing that the vertical distance between effectors’ centers of pressure on the first lift post-transfer was smaller than that of the last lift indicated the latter. Thus, the partial generalization seen when adding or removing effectors is a result of the modulation of effector centers of pressure, specific to the post-transfer grasp type, in the appropriate direction but of insufficient magnitude.

**Table 2:** Characteristics of torque generation before and after transfer. Details of the characteristics of center of pressure (COP) and load force (LF) difference in the pre- and post-transfer lifts. The 4 context switch conditions of the study are shown: (1) adding effectors (unimanual to bimanual) for an object with a left center of mass, (2) adding effectors (unimanual to bimanual) for an object with a right center of mass, (3) removing effectors (bimanual to unimanual) for an object with a left center of mass, (4) removing effectors (bimanual to unimanual) for an object with a right center of mass.

Condition	Unimanual to bimanual		Bimanual to unimanual	
	Pre-transfer unimanual	Post-transfer bimanual	Pre-transfer bimanual	Post-transfer unimanual
**Left center of mass**				
Difference	Higher left side (thumb) COP	Higher left hand COP	Higher left hand COP	Higher left side (thumb) COP
	Larger right side (finger) LF	No LF difference	Larger left hand LF	Larger right side (finger) LF
COP magnitude		Smaller difference than lift 10 Partial transfer		Smaller difference than lift 10 Partial transfer
**Right center of mass**				
Difference	Collinear COP	Higher right hand COP	Higher right hand COP	Higher left side (thumb) COP
	Larger right side (finger) LF	No LF difference	Larger right hand LF	Larger right side (finger) LF
COP magnitude		Smaller difference than lift 10 Partial transfer		Larger difference than lift 10 No transfer

### Effect of center of pressure modulation on generalization after adding or removing effectors

In all instances of partial transfer, this was driven by the modulation of effector centers of pressure, which we suggest also provides an explanation for partial generalization after bimanual transfer regardless of center of mass and after unimanual transfer with a left center of mass. As detailed in the section above, center of pressure modulation during bimanual grasps assists torque generation regardless of center of mass. For unimanual grasps, when the center of mass is on the thumb side, the higher thumb center of pressure modulation assists compensatory torque generation while larger finger load forces resists torque generation (Fig. 8, left COM). When the center of mass is on the side of the fingers, the larger finger load force assists torque generation while collinear centers of pressure significantly reduce the contribution of grip force to the compensatory torque (Fig. 8, right COM). Thus, since transfer is driven by center of pressure modulation, partial transfer is seen only when center of pressure assists torque generation, i.e., both centers of mass after bimanual transfer and the left center of mass after unimanual transfer. Additionally, since bimanual center of pressure characteristics are similar to unimanual characteristics for the left center of mass, this similarity would have aided the partial generalization. It should be noted that aside from the thumb during unimanual lifts, motion capture data did not reveal modulation of individual digit placements for both unimanual and bimanual grasps. Thus, center of pressure modulation was mainly due to the control of individual digit grip forces more than digit placement, when multiple digits are involved. This is similar to our previous finding during whole hand grasping where digit placement modulation was driven by thumb placement^15^. We discuss this further in the section on limitations and future work.

The partial generalization seen in this study was due to the partial modulation of center of pressure and the lack of load force modulation in the direction of torque generation. This is similar to a previous study that showed that the learning of torque generation through multiple 180° object rotations was driven by digit center of pressure^23^. It should also be noted that digit position can be monitored through vision while digit forces cannot. Other studies have shown distinct sensorimotor memories between the different aspects of torque generation^24,25^. However, this result is different from the initial study showing full generalization when adding or removing one digit^17^. A possible explanation for this would be that, unlike the addition or removal of one degree of freedom from two- to three-digit grasping, addition or removal of an entire effector added complexity to the transfer condition. Additionally, the different characteristics of torque generation between unimanual and bimanual grasps appear to be an additional factor influencing the effectors’ center of pressure and load force modulation underlying partial generalization. It should be noted that our study employed unconstrained grasping on both surfaces. Therefore, our results seen cannot be attributed to biomechanical artifacts that could have been caused by forcing participants to place their thumb/digits at constrained positions.

That load forces were not modulated after transfer together with effector center of pressure to generate a compensatory torque indicates weak or absent covariation between the two measures. It is unlikely that this is due to any error in the perception of effector center of pressure and thus we suggest that this is an intentional feature of the system to prevent larger errors when the grasp context changes. We have previously shown that when faced with conflicting sensory cues, grasp characteristics follow collinear torque generation, i.e. zero torque^9^. Thus, when faced with the uncertainty of the changing grasp context, load forces are modulated similar to lifts requiring no net torque to reduce the error should the center of pressure modulation be wrong.

### Theoretical frameworks for generalization across multiple effectors

Generalization of learned movement can also be examined based on the concept of coordinate frames/systems. Specifically, coordinate reference frames refer to intrinsic systems as those associated with the coordinate frame of the body (joint/body-based) while extrinsic systems comprise the dynamics of the object (object/environment-based)^26–29^. Recent work suggests that learning within a task may occur in both coordinate systems^30–33^. It has been suggested that when learning a movement, kinematic trajectories are represented extrinsically while forces are represented intrinsically^30^. In the present study, aside from the center of pressure of the thumb in unimanual grasps, center of pressure modulation was achieved through grip force modulation of the individual fingers as opposed to the actual change in digit placement. Although both grip forces and digit centers of pressure interact to produce the normal component of torque, most studies to date have focused on center of pressure modulation in regard to torque generation. However, our results show that during multi-digit grasping, the interaction of grip force and digit placement to determine the overall center of pressure is important in determining generalization. Though nontrivial, the partial generalization shown here might indicate learning of overall center of pressure in extrinsic coordinates. Further studies would be needed to determine this. In contrast, since load forces are represented intrinsically, their learning is specific to one grasp type and switching to a new grasp does not result in full transfer. An additional explanation could be the reduction of kinematic errors through organizing control points on the object^34^.

Aside from coordinate frames, an aspect of sensorimotor control that may influence generalizability is related to the use of sensory cues. Prior to contact with the object, during the reach-to-grasp phase, digit kinematics, assisted by visual and proprioceptive cues, are molded to the contours of the object’s shape^4,8,35^. Additionally, previous studies have shown that digit forces are controlled and modulated once sensory feedback of digit placement is obtained after object contact^36–38^. Because digit center of pressure can be sensed from visual, tactile, and proprioceptive cues (even in the event of an erroneous efference copy), it has been suggested that center of pressure and digit placement are controlled explicitly. Load forces, being modulated through feedback of digit center of pressure are controlled more implicitly. This distinction could be an additional explanation for the transfer of digit center of pressure but not load forces.

### Limitations and future work

Aside from the thumb during unimanual grasps, our study examined the combined forces and equivalent center of pressure of all the digits. Although the digits work synergistically to generate the compensatory torques, examining individual digit forces and centers of pressure would provide more insight into the control of the kinetics and kinematics of grasping. This could be achieved through subsequent studies that are able to examine individual digit forces and placements while also keeping the grasping unconstrained. Additionally, even though the purpose of the study was to examine dexterous manipulation, the functionality of the bimanual grasp may be limited. When interacting with a similar object, a full palmar grasp might be preferred. We chose to keep the grasp points isolated on the distal fingertips to maintain similarity between unimanual and bimanual grasping, but the functional aspect of changing the grasp type might be affected. Our decision on the degrees of freedom to use was strongly determined by the properties of the object to be grasped such as its width and mass^39,40^. For the present study, the grip width and mass of the object favored five-digit and two-hand grasp configurations allowing the examination of generalization between a unimanual and bimanual grasp. Additionally, by only using the fingertips, this generalization can be explored within the characteristics of a dexterous precision grasp^41,42^. However, whether or not there is any difference in these two types of bimanual grasping remains unclear. Even though we found no significant changes in digit placement for bimanual grasps, it is possible that individual digits were modulated but that the modulation was too small for a difference to be detected. This does not however rule out the possibility that these small modulations together contributed to center of pressure modulation. Perhaps studies employing more sensitive measurements of digit placement might reveal more detail. The difference in learned manipulation between five-digit and bimanual grasping, compared to the similarities between two-digit and bimanual grasping, beg the question of whether generalization would be different in the latter instance. The unique characteristic of five-digit grasping where the fingers always exert larger load forces is an interesting finding that we have shown here and in a previous study^15^. We have suggested that this could be due to a biomechanical constraint of five-digit grasping. Further studies exploring this characteristic of whole-hand unimanual grasping could provide more insight. Examining the results of unimanual load force difference after transfer show a possible trend in the appropriate direction for the right center of mass (Fig. 8). Because load force difference did not show any statistical difference between novel and transfer lifts (Fig. 6), we have made a conservative conclusion that load forces were not affected by transfer. However, it is possible that certain experimental modifications, such as changing the object torque, might illicit load force modulations.

In summary, we found evidence that partial generalization of learned manipulation between unimanual and bimanual grasping is due to the ability to use the representation of learned torque to drive the transfer of center of pressure modulation to generate a compensatory torque in the direction to counter object roll but of insufficient magnitude. Transferring to a grasp context where effector center of pressure has a minimal effect on torque generation (unimanual grasp of an object with center of mass on the side of the fingers) would not lead to transfer. Thus, we conclude that a high-level internal representation of learned compensatory torque can be used to drive partial generalization between unimanual and bimanual grasps, and vice versa through the modulation of effector centers of pressure, but only under certain conditions.

## Materials and methods

### Participants

Twenty healthy adults (median age: 26 years, range 19–34 years; 11 females) with normal or corrected-to-normal vision and no upper limb orthopedic impairments were recruited to participate in the study. Participants were right-handed with handedness determined using the Edinburgh Handedness Inventory (laterality quotient > 90). Written informed consent was obtained prior to participation in compliance with the Declaration of Helsinki. The study was approved by the Teachers College, Columbia University Institutional Review Board.

### Apparatus

For the object, we used a custom-made device similar to the one used in our previous study^15^. Figure 1A shows the schematic of the box (height, width, depth = 16.5, 8.1, 8.5 cm). The box is visually symmetrical with compartments within the box to change its center of mass. Adding lead weights to the left or right compartment generated object torques of − 20 and + 20 Ncm, respectively. Sandpaper (100 grit) covered the carbon fiber grip surfaces on either side (height, width, thickness = 15, 8, 0.3 cm). 6-axis force transducers (Mini 40, ATI Industrial Automation, NC, USA) were situated under the grip surfaces. These force transducers measured grip and load forces, as well as the torque exerted on the surface with a resolution of 0.02 N, 0.01 N, and 0.125 Nmm respectively. An electromagnetic sensor (Polhemus Fastrak, 0.005 mm range, 0.025° resolution) was attached to the top of the device to measure vertical distance and object roll. 3D motion capture was achieved through a Vicon Nexus system (Lake Forest, CA) using 10 Vicon Vero cameras with a resolution of 2048 × 1088 megapixels.

### Procedure

10 participants were assigned to condition 1 while another 10 participants performed lifts according to the order in condition 2. This was done to prevent any potential learning effects within the grasp types. Prior to the start of the experiment, reflective markers were attached to the fingernails of the participants to enable motion capture. Participants were seated in front of a height-adjustable table, with their elbows flexed 90° in the parasagittal plane. Their hands were placed on the edge of the table. They then performed lifts of the box according to the order prescribed in Table 1. All unimanual grasps were performed with the right hand. Participants were instructed to lift the box at a smooth and self-selected pace, with the goal of minimizing the roll of the box. After an audio tone, participants reached and grasped the object on the lateral surfaces with the appropriate grasp type, anywhere on the respective grip surfaces, and lifted the object vertically to align the bottom of the object with a 10 cm reference marker. The object was held at that height until presentation of a second audio tone (5 s after first tone), after which they placed the object back on the table and returned their hands to the start point awaiting the start of the next lift. After the first block of 10 consecutive lifts (novel block), participants were instructed to lift the box with the next grasp type for another 10 consecutive lifts (transfer block). Once participants completed both blocks, they were given a 5-min rest after which, the experiment was repeated with the opposite center of mass in the same transfer condition. The order of center of mass was counterbalanced across participants. Thus, each participant performed 20 trials in the left center of mass, and 20 trials in the right center of mass. This was similar to a previous study^17^. To check for any order effects, we performed a mixed measures ANOVA with hand and center of mass as the within-subjects factor and order as the between-subjects factor on the first lifts. We did not find any effect of order in either condition (*p*’s > 0.05). The first and last lifts in each block were used in the analysis.

### Data processing

Throughout the lifts, effector forces and torques applied to the grip surfaces recorded by the force transducers, and position data of the box recorded by the electromagnetic sensor were sampled at 500 and 120 Hz, respectively, using custom written software in WinSC/Zoom (Umeå University, Sweden). Digit placement data was sampled at 120 Hz. A second-order low pass Butterworth filter with a cutoff frequency of 6 Hz was used to filter the data collected. To examine anticipatory control of digit forces and position, these variables were analyzed at lift-onset, before information signaling the center of mass (COM) location could influence grasp control^11^. Lift onset was defined as the point at which the vertical position of the object went above 1 mm and increased continuously. To further affirm that at the point of lift-off, participants were not using sensory feedback of object center of mass, lift velocities were examined to ensure there were no slow or hesitant lifts. Figure 1B shows how the digit forces and placement are applied in a unimanual and bimanual grasp. The outcome measures included:

1. Peak object roll, defined as the angle of the object in the frontal plane. Peak object roll was recorded within 250 ms after lift onset. It denotes the participants’ ability to accomplish the task goal (object roll minimization). Positive values represent counterclockwise roll (towards the left) and negative values represent clockwise roll (towards the right) (Fig. 1B).

Measures recorded at lift onset:

2. Load force (LF), measured in Newtons (N), is the tangential component of the force exerted on each grasp surface.
  - Load force difference *(LF*_*diff*_*)* = *LF*_*left*_* – LF*_*right*_
    Positive values indicate larger left than right side LF, while negative values indicate larger right than left side LF. It should be noted that using this formula, a positive value for the right hand indicates larger thumb LF.
3. Grip force (GF), measured in Newtons (N), is the average normal component of the force exerted by the digits on each grasp surface. Considering no lateral movement of the object, GF on the left side is equal magnitude and opposite direction to GF on the right side.
4. Center of pressure (COP), measured in centimeters (cm), is the equivalent vertical point of application of all the digits on each grasp surface. For multiple digits, this is the net COP after considering all individual digit COPs. This was computed using the formula^2,23^:
  $$COP_{side} = \left[ {Tx_{side} {-}\left( {LF_{side} * \, width \, of \, grip \, surface} \right)} \right]/GF_{side}$$
  where *Tx*, torque applied in the frontal plane, is the torque generated on each side of the grasp surface measured in Newton centimeter (Ncm). The thickness of the grip surface was 0.5 cm.
  - $${\text{Center }}\;{\text{of}}\;{\text{ pressure}}\;{\text{ difference}}\;(COP_{diff} ) = COP_{left} {-}COP_{right}$$
    Positive values indicate higher left than right side COP, while negative values indicated higher right than left side COP. Similar to LF_diff_, a positive value for the right hand indicates higher thumb COP.
5. Compensatory torque (Tcom), measured in Newtons centimeter (Ncm), was defined as the anticipatory torque generated by the hand/s, to counter object torque. This was computed using a similar formula^2, 23,43^:
  $$Tcom = \left[ {\left( {LF_{diff} } \right) \, * \, d/2} \right] + \left[ {\left( {GF_{average} * \, COP_{diff} } \right)} \right]$$
  where *d* is the width of the box (8.1 cm). A positive Tcom denotes a clockwise torque while a negative Tcom denotes a counter-clockwise torque.
6. Digit placement (DP) is the relative height of all the digits to the bottom of the box and was measured using Vicon motion capture. This allowed us to capture the individual digit placements while the force sensors provided the overall net COP^16^.

### Data analysis

In our previous work, we have shown that the applied Tcom negatively correlates with the resultant object peak roll and is thus a valid measure of performance of the task goal^2,44,45^. Similarly, for our study, we found a strong linear correlation between Tcom and peak roll for all grasp types with a Pearson’s correlation coefficient between − 0.79 and − 0.91 (data from the first and last lift of each participant in each grasp type). Thus, we used Tcom at lift onset in our analysis as the performance variable for each grasp condition. Anticipatory planning of digit forces and placement were further analyzed using the resultant LF_diff_, GF, and COP_diff_ at lift onset. Peak roll, Tcom, GF, LF, and COP were extracted using a custom written software in WinZoom (Umeå University, Sweden). Digit placement was analyzed using Vicon Nexus (Lake Forest, CA).

To determine if adding or removing effectors affected generalization, we performed a mixed-measures ANOVA with trial (trial 1, trial 10, trial 11, trial 20), and COM (left, right) as the within-subjects factor, and condition (unimanual to bimanual, bimanual to unimanual) as the between-subjects factor for Tcom, COP_diff_, LF_diff_, and average GF for each grasp type. To further quantify the respective contribution of the two torque components of Tcom (a tangential torque generated by LF_diff_ and a normal torque generated by COP_diff_ × GF) across each condition before and after transfer, we ran the same mixed-measures ANOVA on the torque generated by each of these two components. To compare digit placement across the lifts, we performed a mixed-measures ANOVA with digit (thumb, index, middle, ring, little finger), lift (first and last), and COM (left and right) as the within-subjects factors and block (novel, transfer) as the between-subjects factor for the unimanual grasp. For the bimanual grasp, to compare the digits of the left and right hand, we added another within-subjects factor of hand (left hand, right hand). Shapiro–Wilk’s normality tests on the individual variables revealed that of the 5 variables (Tcom, COP_diff_, LF_diff_, GF, GFxCOP_diff_), 12 out of the 80 combinations of factors did not meet the assumption of normality. For these variables, we also performed Friedman tests on trial and COM with Wilcoxon signed-rank tests for the post hoc analysis and a Kruskal–Wallis test on (between-subjects) condition. The results of these nonparametric tests were the same as the mixed ANOVA results. As such, we report the results from the mixed ANOVAs. We report effect sizes using partial eta squared, η_p_^2^. Bonferroni corrections were used where applicable. Sphericity assumptions were also tested and corrected using the Greenhouse–Geisser correction where appropriate. Significance was considered at the *p* < 0.05 level. Post-hoc tests, with Bonferroni adjustments, were performed on interactions that reached significance. Details of the results of the statistical analyses can be found in the supplementary table 1 for the mixed ANOVA, and supplementary Table 2 for the nonparametric tests.

## Supplementary Information

Supplementary Information 1.

Supplementary Information 2.

## Data Availability

The data collected for this study are available on request through the corresponding author.

## References

[1] Cohen RG, Rosenbaum DA (2004). Where grasps are made reveals how grasps are planned: generation and recall of motor plans. Exp. Brain Res..

[2] Fu Q, Zhang W, Santello M (2010). Anticipatory planning and control of grasp positions and forces for dexterous two-digit manipulation. J. Neurosci..

[3] Jeannerod, M. Grasping. A historical perspective. *Sensorimotor Control of Grasping: Physiology and Pathophysiology*, 127 (2009).

[4] Santello M, Soechting JF (1998). Gradual molding of the hand to object contours. J. Neurophysiol..

[5] Santello, M. Dexterous manipulation: Bridging the gap between hand kinematics and kinetics. In* Reach-to-Grasp Behavior: Brain, Behavior, and Modelling Across the Life Span* 256–277 (Taylor and Francis, 2018).

[6] Schneider TR, Buckingham G, Hermsdörfer J (2019). Torque planning errors affect the perception of object properties and sensorimotor memories during object manipulation in uncertain grasp situations. J. Neurophysiol..

[7] Schneider TR, Buckingham G, Hermsdörfer J (2020). Visual cues, expectations, and sensorimotor memories in the prediction and perception of object dynamics during manipulation. Exp. Brain Res..

[8] Winges SA, Weber DJ, Santello M (2003). The role of vision on hand preshaping during reach to grasp. Exp. Brain Res..

[9] Lee-Miller T, Marneweck M, Santello M, Gordon AM (2016). Visual cues of object properties differentially affect anticipatory planning of digit forces and placement. PLoS ONE.

[10] Johansson RS, Backlin JL, Burstedt MK (1999). Control of grasp stability during pronation and supination movements. Exp. Brain Res..

[11] Salimi I, Hollender I, Frazier W, Gordon AM (2000). Specificity of internal representations underlying grasping. J. Neurophysiol..

[12] Wing AM, Lederman SJ (1998). Anticipatory load torques produced by voluntary movements. J. Exp. Psychol. Hum. Percept. Perform..

[13] Lukos JR, Ansuini C, Santello M (2008). Anticipatory control of grasping: independence of sensorimotor memories for kinematics and kinetics. J. Neurosci..

[14] Lukos JR, Choi JY, Santello M (2013). Grasping uncertainty: effects of sensorimotor memories on high-level planning of dexterous manipulation. J. Neurophysiol..

[15] Marneweck M, Lee-Miller T, Santello M, Gordon AM (2016). Digit position and forces covary during anticipatory control of whole-hand manipulation. Front. Hum. Neurosci..

[16] Lee-Miller T, Gordon AM, Santello M (2019). Hand forces and placement are modulated and covary during anticipatory control of bimanual manipulation. J. Neurophysiol..

[17] Fu Q, Hasan Z, Santello M (2011). Transfer of learned manipulation following changes in degrees of freedom. J. Neurosci..

[18] Bursztyn LL, Flanagan JR (2008). Sensorimotor memory of weight asymmetry in object manipulation. Exp. Brain Res..

[19] Fu Q, Choi JY, Gordon AM, Jesunathadas M, Santello M (2014). Learned manipulation at unconstrained contacts does not transfer across hands. PLoS ONE.

[20] Gordon, A.M. & Salimi, I. Internal models underlying fingertip force control during object manipulation in humans. In *Conference Proceedings: ... Annual International Conference of the IEEE Engineering in Medicine and Biology Society. IEEE Engineering in Medicine and Biology Society. Annual Conference* **6**, 4641–4644. 10.1109/iembs.2004.1404286 (2004). 10.1109/IEMBS.2004.1404286 17271342

[21] Albert F, Santello M, Gordon AM (2009). Sensorimotor memory of object weight distribution during multidigit grasp. Neurosci. Lett..

[22] Marneweck M, Knelange E, Lee-Miller T, Santello M, Gordon AM (2015). Generalization of dexterous manipulation is sensitive to the frame of reference in which it is learned. PLoS ONE.

[23] Zhang W, Gordon AM, Fu Q, Santello M (2010). Manipulation after object rotation reveals independent sensorimotor memory representations of digit positions and forces. J. Neurophysiol..

[24] Cole KJ, Potash M, Peterson C (2008). Failure to disrupt the ‘sensorimotor’memory for lifting objects with a precision grip. Exp. Brain Res..

[25] Quaney BM, Rotella DL, Peterson C, Cole KJ (2003). Sensorimotor memory for fingertip forces: evidence for a task-independent motor memory. J. Neurosci..

[26] Criscimagna-Hemminger SE, Donchin O, Gazzaniga MS, Shadmehr R (2003). Learned dynamics of reaching movements generalize from dominant to non-dominant arm. J. Neurophysiol..

[27] Krakauer JW, Ghilardi M-F, Ghez C (1999). Independent learning of internal models for kinematic and dynamic control of reaching. Nat. Neurosci..

[28] Krakauer JW, Pine ZM, Ghilardi M-F, Ghez C (2000). Learning of visuomotor transformations for vectorial planning of reaching trajectories. J. Neurosci..

[29] Shadmehr R, Moussavi ZM (2000). Spatial generalization from learning dynamics of reaching movements. J. Neurosci..

[30] Bays PM, Wolpert DM (2006). Actions and consequences in bimanual interaction are represented in different coordinate systems. J. Neurosci..

[31] Berniker M, Franklin DW, Flanagan JR, Wolpert DM, Kording K (2013). Motor learning of novel dynamics is not represented in a single global coordinate system: evaluation of mixed coordinate representations and local learning. J. Neurophysiol..

[32] Berniker M, Kording K (2008). Estimating the sources of motor errors for adaptation and generalization. Nat. Neurosci..

[33] Brayanov JB, Press DZ, Smith MA (2012). Motor memory is encoded as a gain-field combination of intrinsic and extrinsic action representations. J. Neurosci..

[34] Heald JB, Ingram JN, Flanagan JR, Wolpert DM (2018). Multiple motor memories are learned to control different points on a tool. Nat. Hum. Behav..

[35] Santello M, Soechting JF (1997). Matching object size by controlling finger span and hand shape. Somatosens. Mot. Res..

[36] Mojtahedi K, Fu Q, Santello M (2015). Extraction of time and frequency features from grip force rates during dexterous manipulation. IEEE Trans. Biomed. Eng..

[37] Shibata D, Santello M (2017). Role of digit placement control in sensorimotor transformations for dexterous manipulation. J. Neurophysiol..

[38] Toma S, Shibata D, Chinello F, Prattichizzo D, Santello M (2019). Linear integration of tactile and non-tactile inputs mediates estimation of fingertip relative position. Front. Neurosci..

[39] Cesari P, Newell KM (1999). The scaling of human grip configurations. J. Exp. Psychol. Hum. Percept. Perform..

[40] Cesari P, Newell KM (2000). Body-scaled transitions in human grip configurations. J. Exp. Psychol. Hum. Percept. Perform..

[41] Feix, T., Pawlik, R., Schmiedmayer, H.-B., Romero, J. & Kragic, D. in *Robotics, Science and Systems: Workshop on Understanding the Human Hand for Advancing Robotic Manipulation.* 2.3.

[42] Feix T, Romero J, Schmiedmayer H-B, Dollar AM, Kragic D (2015). The grasp taxonomy of human grasp types. IEEE Trans. Hum. Mach. Syst..

[43] Latash ML, Zatsiorsky VM (2009). Multi-finger prehension: control of a redundant mechanical system. Adv. Exp. Med. Biol..

[44] Fu Q, Santello M (2012). Context-dependent learning interferes with visuomotor transformations for manipulation planning. J. Neurosci..

[45] Fu Q, Santello M (2014). Coordination between digit forces and positions: interactions between anticipatory and feedback control. J. Neurophysiol..

