# Supplementary material for: Transfer and generalization of learned manipulation between unimanual and bimanual tasks
Source: Sci Rep. 2021 Apr 22;11:8688. doi: 10.1038/s41598-021-87988-0 (PMC8062521; doi:10.1038/s41598-021-87988-0)
Supplement: Supplementary file 1 — Supplementary Information 1. [file 41598_2021_87988_MOESM1_ESM.pdf]

Supplementary Table 1: Results of the mixed ANOVA on the main variables

| | Within-Subjects Main effects | | Between-Subjects Effect | Interactions | | | |
|---|---|---|---|---|---|---|---|
| Measure | Trial | COM | Condition | Trial*COM | Trial*Condition | COM*Condition | Trial*COM*Condition |
| Tcom | $F(3,54) = 1.38$<br>$p = .26$<br>$\eta_p^2 = 0.07$ | $F(1,18) = 3072.78$<br>$p < .001$<br>$\eta_p^2 = 0.99$ | $F(1,18) = 0.72$<br>$p = .41$<br>$\eta_p^2 = 0.04$ | $F(3,54) = 310.69$<br>$p < .001$<br>$\eta_p^2 = 0.95$ | $F(3,54) = 0.84$<br>$p = .47$<br>$\eta_p^2 = 0.05$ | $F(1,18) = 14.40$<br>$p = .001$<br>$\eta_p^2 = 0.44$ | $F(3,54) = 36.18$<br>$p < .001$<br>$\eta_p^2 = 0.67$ |
| $COP_{diff}$ | $F(2.03,36.58) = 19.92$<br>$p < .001$<br>$\eta_p^2 = 0.53$ | $F(1,18) = 231.36$<br>$p < .001$<br>$\eta_p^2 = 0.93$ | $F(1,18) = 0.54$<br>$p = .47$<br>$\eta_p^2 = 0.03$ | $F(3,54) = 67.47$<br>$p < .001$<br>$\eta_p^2 = 0.79$ | $F(3,54) = 2.31$<br>$p = .09$<br>$\eta_p^2 = 0.11$ | $F(1,18) = 6.78$<br>$p = .02$<br>$\eta_p^2 = 0.27$ | $F(3,54) = 7.33$<br>$p < .001$<br>$\eta_p^2 = 0.29$ |
| $LF_{diff}$ | $F(2.04,36.66) = 31.18$<br>$p < .001$<br>$\eta_p^2 = 0.63$ | $F(1,18) = 45.01$<br>$p < .001$<br>$\eta_p^2 = 0.71$ | $F(1,18) = 1.83$<br>$p = .19$<br>$\eta_p^2 = 0.09$ | $F(3,54) = 9.81$<br>$p < .001$<br>$\eta_p^2 = 0.35$ | $F(3,54) = 2.61$<br>$p = .06$<br>$\eta_p^2 = 0.13$ | $F(1,18) = 0.57$<br>$p = .46$<br>$\eta_p^2 = 0.03$ | $F(3,54) = 1.81$<br>$p = .17$<br>$\eta_p^2 = 0.09$ |
| GF | $F(1.74,31.30) = 58.80$<br>$p < .001$<br>$\eta_p^2 = 0.77$ | $F(1,18) = 0.002$<br>$p = .96$<br>$\eta_p^2 = 0.0001$ | $F(1,18) = 0.027$<br>$p = .87$<br>$\eta_p^2 = 0.002$ | $F(1.96,35.24) = 2.17$<br>$p = .13$<br>$\eta_p^2 = 0.11$ | $F(3,54) = 5.94$<br>$p = .001$<br>$\eta_p^2 = 0.25$ | $F(1,18) = 0.10$<br>$p = .75$<br>$\eta_p^2 = 0.006$ | $F(3,54) = 0.04$<br>$p = .99$<br>$\eta_p^2 = 0.002$ |

Tcom = Compensatory Torque, $COP_{diff}$ = Center of pressure difference, $LF_{diff}$ = Load force difference, GF = Grip force
