# Supplementary material for: Transfer and generalization of learned manipulation between unimanual and bimanual tasks
Source: Sci Rep. 2021 Apr 22;11:8688. doi: 10.1038/s41598-021-87988-0 (PMC8062521; doi:10.1038/s41598-021-87988-0)
Supplement: Supplementary file 2 — Supplementary Information 2. [file 41598_2021_87988_MOESM2_ESM.pdf]

Supplementary Table 2: Results of the nonparametric tests on the main variables

Friedman Test (Trial)

	Condition 1		Condition 2	
	Left center of mass (LCM)	Right center of mass (RCM)	Left center of mass (LCM)	Right center of mass (RCM)
Tcom	$\chi^2(3) = 27.48, p < .001$	$\chi^2(3) = 28.08, p < .001$	$\chi^2(3) = 23.40, p < .001$	$\chi^2(3) = 25.20, p < .001$
COP_{diff}	$\chi^2(3) = 18.84, p < .001$	$\chi^2(3) = 24.00, p < .001$	$\chi^2(3) = 16.80, p = .001$	$\chi^2(3) = 21.24, p < .001$
LF_{diff}	$\chi^2(3) = 14.15, p = .003$	$\chi^2(3) = 7.68, p = .053$	$\chi^2(3) = 20.76, p < .001$	$\chi^2(3) = 23.46, p < .001$
GF	$\chi^2(3) = 9.48, p = .024$	$\chi^2(3) = 19.56, p < .001$	$\chi^2(3) = 25.32, p < .001$	$\chi^2(3) = 27.48, p < .001$

Wilcoxon Signed Rank Test

	Trial 1 - Trial 10				Trial 10 - Trial 11				Trial 11 - Trial 20			
	Condition 1		Condition 2		Condition 1		Condition 2		Condition 1		Condition 2	
	LCM	RCM	LCM	RCM	LCM	RCM	LCM	RCM	LCM	RCM	LCM	RCM
Tcom	$p = .005$	$p = .005$	$p = .005$	$p = .005$	$p = .005$	$p = .005$	$p = .007$	$p = .005$	$p = .005$	$p = .005$	$p = .005$	$p = .005$
COP_{diff}	$p = .005$	$p = .005$	$p = .005$	$p = .005$	$p = .013$	$p = .074$	$p = .33$	$p = .005$	$p = .009$	$p = .028$	$p = .24$	$p = .017$
LF_{diff}	$p = .96$	$p = .022$	$p = .005$	$p = .028$	$p = .059$	$p = .059$	$p = .005$	$p = .019$	$p = .52$	$p = .11$	$p = .17$	$p = .017$
GF	$p = .037$	$p = .005$	$p = .005$	$p = .005$	$p = .017$	$p = .005$	$p = .007$	$p = .005$	$p = .88$	$p = .45$	$p = .51$	$p = .059$

	LCM - RCM (Condition 1)				LCM - RCM (Condition 2)			
	Unimanual		Bimanual		Bimanual		Unimanual	
	Trial 1	Trial 10	Trial 11	Trial 20	Trial 1	Trial 10	Trial 11	Trial 20
Tcom	$p = .017$	$p = .005$	$p = .005$	$p = .005$	$p = .017$	$p = .005$	$p = .005$	$p = .005$
COP_{diff}	$p = .29$	$p = .005$	$p = .005$	$p = .005$	$p = .17$	$p = .005$	$p = .007$	$p = .007$
LF_{diff}	$p = .80$	$p = .037$	$p = .059$	$p = .005$	$p = .28$	$p = .005$	$p = .51$	$p = .005$
GF	$p = .72$	$p = .72$	$p = .51$	$p = .89$	$p = .65$	$p = .58$	$p = .33$	$p = .11$

Tcom = Compensatory Torque
COP_{diff} = Center of pressure difference
LF_{diff} = Load force difference
GF = Grip force

Kruskal Wallis Test (Condition 1 - Condition 2)

	Bimanual: Novel Trial 1 - Transfer Trial 11		Unimanual: Novel Trial 1 - Transfer Trial 11	
	LCM	RCM	LCM	RCM
Tcom	$\chi^2(1) = 14.29, p < .001$	$\chi^2(1) = 13.16, p < .001$	$\chi^2(1) = 6.22, p = .013$	$\chi^2(1) = 1.85, p = .17$
COP_{diff}	$\chi^2(1) = 11.06, p = .001$	$\chi^2(1) = 4.65, p = .031$	$\chi^2(1) = 4.17, p = .041$	$\chi^2(1) = 0.82, p = .36$
LF_{diff}	$\chi^2(1) = 1.85, p = .17$	$\chi^2(1) = 1.85, p = .17$	$\chi^2(1) = 3.57, p = .059$	$\chi^2(1) = 3.86, p = .050$
GF	$\chi^2(1) = 9.15, p = .002$	$\chi^2(1) = 10.08, p = .001$	$\chi^2(1) = 2.52, p = .11$	$\chi^2(1) = 2.29, p = .13$
